# Profiling gene promoter occupancy of Sox2 in two phenotypically distinct breast cancer cell subsets using chromatin immunoprecipitation and genome-wide promoter microarrays

**DOI:** 10.1186/s13058-014-0470-2

**Published:** 2014-11-08

**Authors:** Karen Jung, Peng Wang, Nidhi Gupta, Keshav Gopal, Fang Wu, Xiaoxia Ye, Abdulraheem Alshareef, Gilbert Bigras, Todd P McMullen, Bassam S Abdulkarim, Raymond Lai

**Affiliations:** 1 Department of Oncology, University of Alberta, Edmonton, T6G 2E1 Alberta Canada; 2 Department of Laboratory Medicine and Pathology, University of Alberta, Edmonton, T6G 2E1 Alberta Canada; 3 Department of Surgery, University of Alberta, Edmonton, T6G 2E1 Alberta Canada; 4 Department of Oncology, McGill University, Montreal, Canada; 5 DynaLIFEDx Medical Laboratories, Edmonton, Canada

## Abstract

**Introduction:**

Aberrant expression of the embryonic stem cell marker Sox2 has been reported in breast cancer (BC). We previously identified two phenotypically distinct BC cell subsets separated based on their differential response to a Sox2 transcription activity reporter, namely the reporter-unresponsive (RU) and the more tumorigenic reporter-responsive (RR) cells. We hypothesized that Sox2, as a transcription factor, contributes to their phenotypic differences by mediating differential gene expression in these two cell subsets.

**Methods:**

We used chromatin immunoprecipitation and a human genome-wide promoter microarray (ChIP-chip) to determine the promoter occupancies of Sox2 in the MCF7 RU and RR breast cancer cell populations. We validated our findings with conventional chromatin immunoprecipitation, quantitative reverse transcription polymerase chain reaction (qPCR), and western blotting using cell lines, and also performed qPCR using patient RU and RR samples.

**Results:**

We found a largely mutually exclusive profile of gene promoters bound by Sox2 between RU and RR cells derived from MCF7 (1830 and 456 genes, respectively, with only 62 overlapping genes). Sox2 was bound to stem cell- and cancer-associated genes in RR cells. Using quantitative RT-PCR, we confirmed that 15 such genes, including *PROM1* (CD133), *BMI1*, *GPR49* (LGR5), and *MUC15*, were expressed significantly higher in RR cells. Using siRNA knockdown or enforced expression of Sox2, we found that Sox2 directly contributes to the higher expression of these genes in RR cells. Mucin-15, a novel Sox2 downstream target in BC, contributes to the mammosphere formation of BC cells. Parallel findings were observed in the RU and RR cells derived from patient samples.

**Conclusions:**

In conclusion, our data supports the model that the Sox2 induces differential gene expression in the two distinct cell subsets in BC, and contributes to their phenotypic differences.

**Electronic supplementary material:**

The online version of this article (doi:10.1186/s13058-014-0470-2) contains supplementary material, which is available to authorized users.

## Introduction

Sex-determining region Y (SRY)-box binding protein-2 (Sox2) is a transcription factor essential to the maintenance of the pluripotent stem cell state in embryonic stem cells (ESCs) and induced pluripotent stem cells [1]-[3]. In human ESCs, Sox2 governs their pluripotency by binding to the promoters of its target genes and transcriptionally regulating their expressions both positively and negatively [2]. A previous study of Sox2 promoter occupancy in human ESCs using chromatin immunoprecipitation promoter microarray chip analysis (ChIP-chip) has revealed target genes positively regulated by Sox2 (including *SOX2*, *OCT4*, *NANOG* and *MYC*) [2]. In normal adult tissues, Sox2 is largely restricted to somatic stem cells; specifically, Sox2 expression has been detected in the stem/progenitor cells of the brain, stomach, colon, and anus [4]. In normal mammary glands, Sox2 is largely restricted to the stem cell populations [5]-[8].

In recent years, Sox2 has been discovered to be aberrantly expressed in cancer cells, including those of the lungs, brain, ovaries, bone, colon, skin, and breasts [8]-[15]. In many of these studies, Sox2 was found in the cancer stem cell population [7],[12],[16]-[22], supporting the hypothesis that cancer stemness is related to the aberrant expression of ESC proteins. It has been demonstrated that Sox2 promotes key tumorigenic properties in cancer cells, including enhanced proliferation, invasion, migration, colony formation, non-adherent stem cell-associated sphere formations *in vitro*, and tumorigenicity *in vivo* [8],[12],[19]-[24]. Further, Sox2 has been shown to correlate with a worse prognosis in cancer patients, including those with breast cancer (BC) [7],[15],[25]-[28]. Up to 30% of BC, including all four major molecular subtypes, have been reported to express Sox2 [7],[8]. In a relatively small number of *in vitro* studies, Sox2 has been directly implicated in promoting cell proliferation, mammosphere formation, invasion and tumorigenesis in BC [7],[8],[29].

We recently identified and characterized two distinct cell subsets of BC, separated based on their differential responsiveness to a Sox2 transcription activity reporter [18]. Using two estrogen receptor-positive (ER+) cell lines, MCF7 and ZR751, we found that the vast majority of these cells, despite robust levels of Sox2, were reporter unresponsive (labeled as RU cells), while a relatively small cell subset were reporter responsive (labeled as RR cells) [18]. Importantly, RU and RR cells are phenotypically distinct, with RR cells showing a higher expression of the stem cell marker CD49f and exhibiting a higher tumorigenic potential [18]. In view of the fact that Sox2 is a transcription factor, we hypothesized that Sox2 mediates differential gene expressions in RU and RR cells, thereby contributing to their phenotypic differences. To test this hypothesis, we analyzed and compared the global promoter occupancy of Sox2 in RU and RR cells using ChIP-chip. As detailed below, we found that the Sox2 gene promoter occupancy between RU and RR cells are mutually exclusive. Importantly, we identified a number of stem cell- or cancer-associated genes that were more highly expressed in RR cells.

## Methods

### Cell lines and materials

MCF7 and ZR751 parental cells were purchased from American Type Culture Collection (ATCC, Rockville, MD, USA). MCF7 and ZR751 parental cells, unsorted cells, RU (previously referred to as GFP Neg), and RR (previously referred to as GFP Pos) cells were cultured and derived as previously described [18]. Triptolide was purchased from Sigma-Aldrich (T3652, Sigma-Aldrich Canada, Oakville, ON, Canada).

### Soxtranscription activity reporter

The commercially available Sox2 transcription activity reporter is driven by a minimal CMV promoter followed by three tandem repeats of the Sox2 regulatory region 2 (SRR2), a sequence containing a Sox2 consensus sequence demonstrated to be bound by Sox2 in mouse and human embryonic stem cells [30].

### ChIP (chromatin immunoprecipitation)-chip and ChIP-PCR

ChIP-chip was performed based on a previously described ChIP-PCR protocol [18]. The starting material was scaled up four times, such that starting materials were four 15-cm plates of both MCF7 RU and RR cells, and four identical immunoprecipitations were performed for each condition (MCF7 RU and RR, IgG and Sox2 IPs). The resulting DNA was further purified using the QIAquick PCR Purification Kit (Qiagen Canada, Toronto, ON, Canada), lyophilized, and reconstituted in 10 μL of UltraPure DNase/RNase-free distilled water (Life Technologies, Burlington, ON, Canada). The DNA was subsequently amplified twice using the Sigma GenomePlex Complete Whole Genome Amplification Kit (#WGA2, Sigma-Aldrich Canada) using a published adapted protocol [31]. ChIP-PCR was performed as previously described [18]. ChIP input DNA was run on an agarose gel to check for microarray optimized DNA fragments of 200 to 1200 bp (Additional file 1: Figure S1A ). DNA samples were sent in two replicates to Roche Nimblegen ChIP-chip Microarray Services for quality assessment, and full service ChIP-chip microarray service and analysis. Briefly, DNA samples were hybridized to the Roche Nimblegen Human ChIP-chip 3x720K RefSeq Promoter array, with promoter tiling ranging from -3,200 to +800 relative to the transcription start site. The ChIP-chip microarray data have been submitted to the public repository Gene Expression Omnibus [GEO: GSE61703]. Primers for ChIP-PCR were designed to flank the promoter peaks identified by ChIP-chip analysis for each gene.

### RNA extraction, cDNA synthesis, quantitative reverse transcription PCR (qPCR)

Total RNA extraction was performed with the Qiagen RNeasy Kit (Qiagen Canada) according to the manufacturer’s protocol: 1 μg of RNA was reverse transcribed using Oligo dT and Superscript II (Life Technologies) according to the manufacturer’s protocol. 1 μL of the resulting cDNA mixture was added to the Platinum SYBR Green qPCR SuperMix-UDG with Rox (Life Technologies) and amplified with target gene-specific primers. Please see Additional file 2: Table S1 for list of PrimerBank primer sequences [32],[33]. All genes of interest are normalized to glyceraldehyde-3-phosphate dehydrogenase (GAPDH) transcript expression levels except for the Triptolide experiments where 18S rRNA was used as the housekeeping gene for its superior stability.

### SiRNA transfections

Sox2 siRNAs (SMARTpool: ON-TARGETplus SOX2 siRNA, Dharmacon, Thermo Scientific, Waltham, MA, USA) or scrambled (Scr) siRNAs (ON-TARGETplus Non-targeting Pool, #477C20, Dharmacon, ThermoScientific) at 40 pmol per rxn (20 nM final concentration) and 5 μL of Lipofectamine RNAiMAX (Life Technologies) were added to 0.5 mL of OptiMEM media (Life Technologies) and reverse transfected to 800,000 cells in normal culture medium in a 6-well plate format. Cells were incubated with siRNAs for 72 hours before harvesting. Muc15 siRNA (#SI04331166, Qiagen Canada, and SMARTpool: ON-TARGETplus MUC15 siRNA, Dharmacon) was transfected in the same manner at 80 pmol and 200 pmol per rxn respectively (40 nM and 100 nM final concentration).

### Western blotting

Western blot analyses were performed as previously described [34]. All antibodies were diluted in 5% BSA in TBST: Sox2 (1:500, #2683-1, Epitomics, Burlingame, CA, USA), FlagM2 (1:1000, #F1804, Sigma-Aldrich), Muc15 (1:500, #ab98045, Abcam, Cambridge, UK), and vinculin (1:1000, #4650, Cell Signaling Technologies, Danvers, MA, USA). Vinculin was used as loading control for all western blots.

### Plasmid transfections

We transfected 3 μg of pcDNA-Flag-EV or pcDNA-Flag-Sox2 with 5 μL of Lipofectamine 2000 (Life Technologies) in 0.5 mL of OptiMEM media (Life Technologies) to 1.2 million MCF7 cells seeded the day before. Cells were incubated for 72 hours before harvesting.

### Mammosphere assay

Mammospheres were generated as previously described [18]. Mammospheres were collected by centrifugation at 300 × g for 5 minutes and trypsinized before subjecting to trypan blue exclusion assay of mammosphere-derived cells.

### Primary patient breast tumor cells isolation, lentiviral infections, fluorescence-activated cell sorting (FACS) purification

Patient material and clinical information were collected with full written consent from the patients and with approval by the University of Alberta Human Research Ethics Board, approved project ID Pro00044942. Fresh breast tumors were collected in cold 100% FBS and harvested within hours. We isolated breast tumor cells from fresh breast tumor tissues with no exposure to radiation therapy or chemotherapy. We harvested purified primary BC cells first by mechanical dissociation and then by using the Cancer Cell Isolation Kit (Panomics Solutions, Affymetrix, Santa Clara, CA, USA) as per manufacturer’s protocol. Cells were cultured in 10% RPMI medium for 48 hours before virus infection. We generated a new dual green fluorescent protein (GFP)/red fluorescent protein (RFP) lentiviral Sox2 reporter by replacing the puromycin resistance gene in the Sox2 reporter with the red fluorescent protein (RFP) gene. Isolated tumor cells were infected with our modified lentiviral Sox2 GFP-RFP dual-color reporter, SRR2-mCMV-GFP-EF1-RFP, twice 24 hours apart. RFP+ cells were gated to include only successfully infected primary breast tumor cells in subsequent analyses and experiments. Using flow cytometry, we analyzed and collected RFP+/GFP- (RU) and RFP+/GFP+ (RR) cells.

### Statistical analyses

The paired Student’s *t*-test was used for statistical analysis of experiments throughout: *P <0.05; **P <0.01.

## Results

### The Sox2-bound gene promoter regions are largely mutually exclusive between RU and RR cells

**Figure 1:**
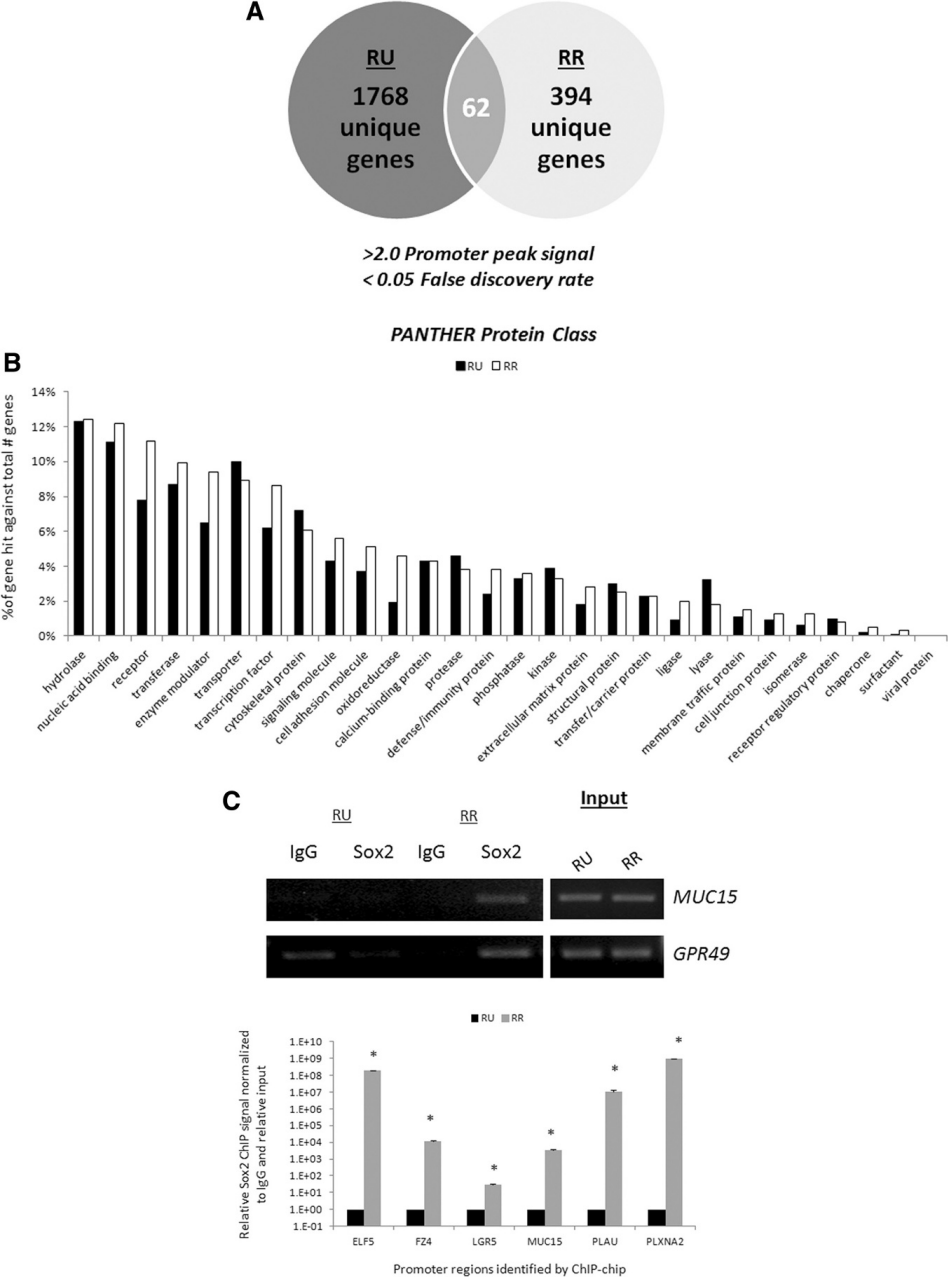
**Sex-determining region Y (SRY)-box binding protein-2 (Sox2) occupies distinct promoter regions in reporter unresponsive (RU) and reporter responsive (RR) breast cancer cells. (A)** Venn diagram of MCF7 RU and RR cells Sox2 chromatin immunoprecipitation promoter microarray chip analysis (ChIP-chip) study summarizing gene promoters bound by Sox2. **(B)** Functional annotation of MCF7 RU and RR putative Sox2 target genes with >2.0 peak score signal (compared to input DNA) using Protein Analysis THrough Evolutionary Relationships (PANTHER) Protein Class system. **(C)** MCF7 RU and RR ChIP DNA agarose gel results of DNA sequences immunoprecipitated by normal rabbit IgG or a rabbit anti-human Sox2 antibody amplified by *GPR49* and *MUC15* promoter specific primers. MCF7 RU and RR input represent the DNA isolated from chromatin before immunoprecipitation to show equal input amounts. Quantitative-PCR analyses of ChIP DNA derived from the IgG and Sox2 ChIP of MCF7 RU and RR cells using promoter-specific primers. Sox2 ChIP-qPCR signal was normalized to IgG signal as well as the respective RU and RR input signal.

Using ChIP-chip, we queried the global promoter occupancy profile of Sox2 in the two phenotypically distinct cell subsets, namely RU and RR cells. Using a stringent threshold (a promoter array peak signal >2.0, compared to the input DNA signal) and a false discovery rate of <0.05, we found that Sox2 was bound to the promoter regions of 1,830 genes in RU cells and 456 genes in RR cells, with an overlap of only 62 genes between the two cell subsets (illustrated in Figure 1A). The complete RU and RR gene lists can be found in Additional file 3: Table S2. ChIP-chip gene promoter analyses are detailed in Additional file 1 (Supplementary materials and methods).

To understand the possible biological effects exerted by Sox2 in BC cells, we annotated the functions of the identified genes using the Protein Analysis THrough Evolutionary Relationships (PANTHER) Protein Class classification system software [35]. As shown in Figure 1B, the biological functions associated with the identified genes are largely similar between the RU and RR cells, with the functions falling most frequently into the categories of hydrolases, nucleic acid binding, and receptors.

### The RR gene list comprise markers associated with cancer stem cells

As we have previously shown that RR cells exhibit more tumorigenic and stem-like properties than RU cells [18] we hypothesized that the ChIP-chip gene list derived from RR cells will contain genes that are known to be associated with cancer stem cells. To test this hypothesis, we searched our RR gene lists for reported cancer stem cell markers, based on those described in two recent publications [36],[37]. We found that Sox2 was bound to the gene promoters of three established stem cell markers in solid tumors, including CD133 (*PROM1*), Lgr5 (*GPR49*), and Bmi-1 (*BMI1*). Importantly, these three genes were not on the RU gene list.

When we examined the remaining 453 genes identified in RR cells, we identified 12 additional genes that have been previously implicated in cancer initiation and/or progression (Table 1). These genes include *FZD4* (the Wnt pathway) [38],[39], *PLAU* (encoding metastasis-promoting protein urokinase plasminogen activator) [40] and *ELF5* (a normal mammary stem/progenitor cell gene) [41]. None of these 12 genes were found in the RU gene list and the majority of these genes (8 of 15) had a very high microarray signal >2.5. Interestingly, *ANTXR1*, also found in our RR gene list, which encodes anthrax toxin receptor-1, has just been recently reported as a stem cell gene important to the tumorigenesis of BC [42],[43]. Again, this gene was not found in the RU gene list.

**Table 1:** **Sox2 interacts with the promoters of stem cell and/or cancer-associated genes in RR cells**

NCBI gene symbol	Description	Final peak score
*PLXNA2*	Plexin A2	3.19
*FZD4*	Frizzled homolog 4 (Drosophila)	3.16
*MUC15*	Mucin 15, cell surface associated	3.04
*PLAU*	Plasminogen activator, urokinase	2.89
ELF5*	E74-like factor 5 (ets domain transcription factor)	2.88
*VCAM1*	Vascular cell adhesion molecule 1	2.68
*DACH2*	Dachshund homolog 2 (Drosophila)	2.55
GPR49*	Leucine-rich repeat-containing G protein-coupled receptor 5	2.55
*FYB*	FYN binding protein	2.43
*COL4A5*	Collagen, type IV, alpha 5	2.40
*MYH9*	Myosin, heavy chain 9, non-muscle	2.39
PROM1 (CD133)*	Prominin 1	2.20
*ESR2*	Estrogen receptor 2 (ER beta)	2.19
*PLAG1*	Pleiomorphic adenoma gene 1	2.19
BMI1*	BMI1 polycomb ring finger oncogene	2.17

List of RR ChIP-chip putative Sox2 target genes. Top-ranked 15 stem cell and/or cancer-associated genes of interest with >2.0 peak score derived from the MCF7 reporter responsive cells and their final adjusted microarray peak scores are summarized. The asterisks denote established stem cell markers.

### Validation of the ChIP-chip data using ChIP-PCR

We then aimed to validate the observation that the gene promoters bound by Sox2 in RU and RR cells are largely non-overlapping. To do so, we employed ChIP-PCR and used two genes from the RR gene list that show relatively high microarray signals and robust mRNA expression in BC cells, namely *GPR49* and *MUC15* [44],[45]. The ChIP-PCR primers for these two genes were designed to flank the exact promoter locations specified by the ChIP-chip microarray probes. As shown in Figure 1C, in RR cells, we detected more robust Sox2 binding at both the *GPR49* and *MUC15* gene promoters than in the RU cells that showed barely detectable to no binding. These ChIP-PCR results support the validity of the ChIP-chip findings. To further validate our ChIP-PCR findings, we also pursued ChIP-qPCR analyses of six gene promoters of interest with high peak scores from the RR gene list, and validated that Sox2 was significantly more frequently bound to these promoters in the RR cells when compared to the RU cells (Figure 1C).

To further test if Sox2 binds to different sets of gene promoters between RU and RR cells, we performed ChIP-PCR to detect the binding of Sox2 to *CCND1* (Cyclin D1) promoter, a direct Sox2 gene target previously shown by us and others [8],[18]. We found the interaction between Sox2 and the *CCND1* gene promoter, but only in RR (data not shown, previously reported by us [18]. We also validated our ChIP DNA by looking at several Sox2 target genes found in human ESCs previously described in the literature, including *BCL2* and *CDH1* [46] As shown in Additional file 1: Figure S1B, we found that Sox2 showed significantly greater binding at the promoters of *BCL2* and *CDH1* in RR cells than in RU cells. Of note, *CCDN1*, *BCL2*, and *CDH1* were not found in our ChIP-chip gene list, likely due to our very stringent analysis criteria, which were used to identify only the most frequently bound DNA sequences in BC cells.

### RR cells express elevated levels of target genes compared to RU cells

**Figure 2:**
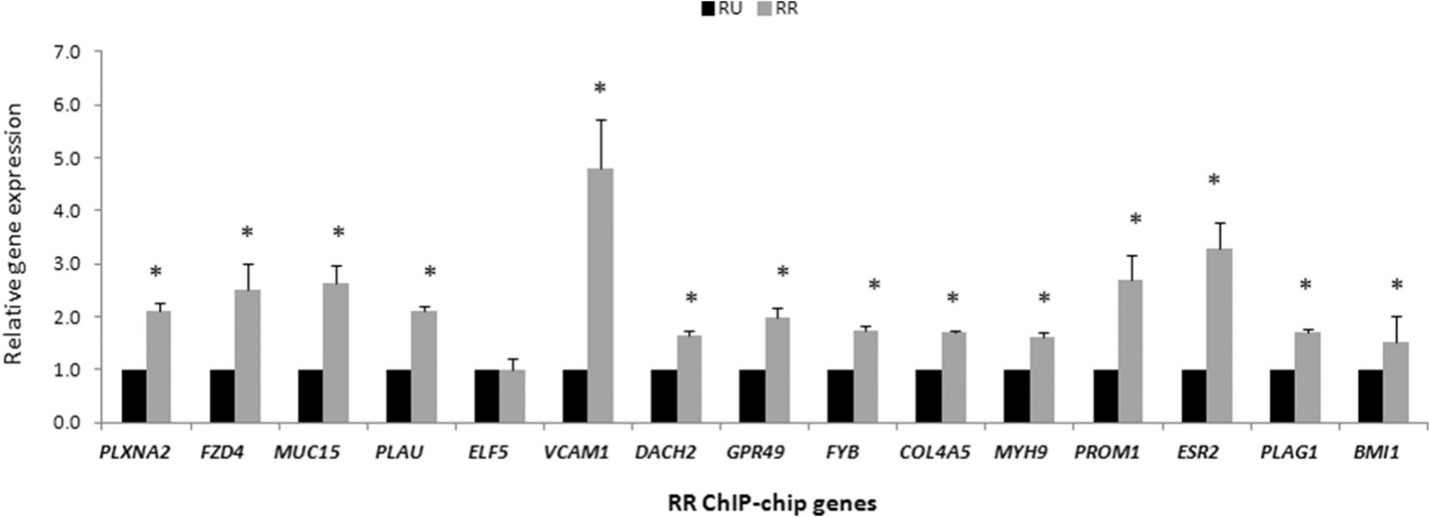
**Reporter responsive (RR) cells exhibit higher expression of target genes.** Quantitative-PCR mRNA transcript analysis of top 15 RR chromatin immunoprecipitation promoter microarray chip analysis (ChIP-chip) genes in MCF7. RU, reporter unresponsive cells.

We next asked if the differential Sox2 gene promoter occupancy between RU and RR cells correlates to significant differences in gene expression between these two cell subsets. Using qRT-PCR, we measured and compared the expression levels of the 15 genes of interest described in Table 1. As compared to RU cells, RR cells expressed significantly higher (2- to 5-fold) gene transcript levels of 14 out of these 15 genes (Figure 2). These results support our hypothesis that Sox2 mediates differential gene expression between RU and RR cells.

### Overexpression of Soxup-regulates target genes in RR cells but not RU cells

**Figure 3:**
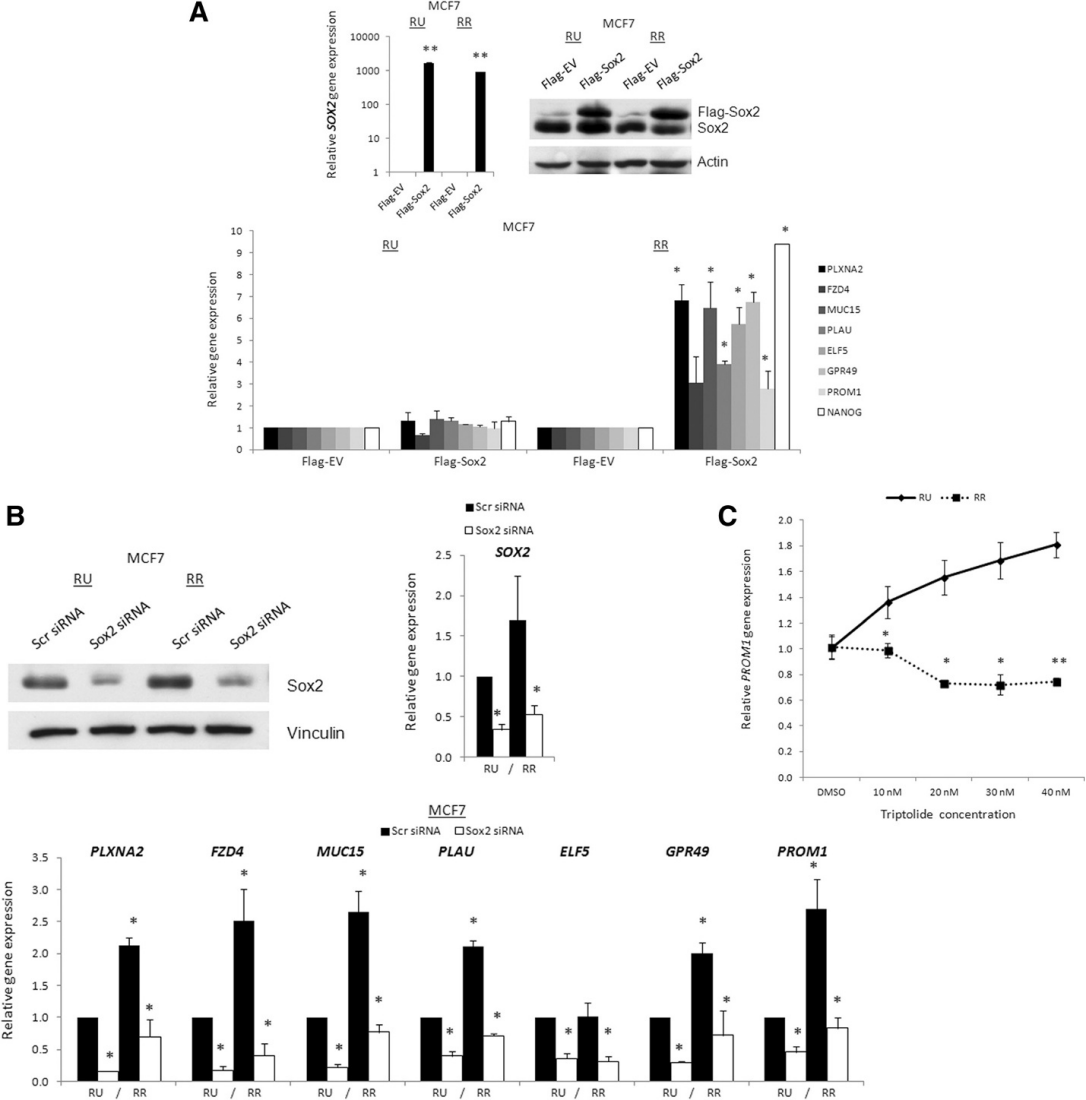
**Sex-determining region Y (SRY)-box binding protein-2 (Sox2) can upregulate target gene transcripts in reporter responsive (RR) cells only and not reporter unresponsive (RU) cells. (A)** MCF7 RU and RR cells were transfected with 3 μg of pcDNA3-Flag-Empty vector (EV), or pcDNA3-Flag-Sox2 (Sox2) and harvested for mRNA after 72 hours. Quantitative (q)-PCR analyses were performed using primers designed against MCF7 RR chromatin immunoprecipitation promoter microarray chip analysis (ChIP-chip) targets. Accompanying Sox2 qPCR analysis and Flag western blot shows transfection efficiency. **(B)** Western blot showing Sox2 knockdown efficiency in MCF7 RU and RR cells after 72-hour 20-nM scrambled or Sox2 siRNA treatment. q-PCR mRNA transcript analysis of MCF7 RU and RR cells after 72-hour 20-nM scrambled or Sox2 siRNA knockdown examining RR ChIP-chip genes in MCF7 RU and RR. **(C)** q-PCR analysis of MCF7 RU and RR cell *PROM1* (CD133) transcripts after 16-hour treatments with dimethyl sulfoxide vehicle control or 10, 20, 30, or 40 nM Triptolide.

To demonstrate the direct role of Sox2 in contributing to the differential gene expression between RU and RR, we examined if enforced expression of Sox2 in MCF7 cells results in significant alterations of their expressions. For the purpose of this study, we chose 7 of the 15 genes, based on their relatively high ChIP-chip peak scores, including *PLXNA2*, *FZD4*, *MUC15*, *PLAU*, *ELF5*, *GPR49* and *PROM1*. As shown in Figure 3A, with transient transfection of *Sox2* into RR cells, all seven genes examined showed a significant increase in their transcript levels in RR cells (3- to 7-fold); conversely, RU cells showed no significant alterations of any of these seven genes.

### SiRNA knockdown of Soxdownregulates target genes

Next, we examined if siRNA knockdown of Sox2 also can modulate the expression of the seven target genes tested. As shown in Figure 3B, the efficiency of the knockdown was demonstrated by western blotting and quantitative RT-PCR. We found that Sox2 siRNAs significantly downregulated these target genes in RR cells. Surprisingly, the same treatment also significantly downregulated the expression of these seven genes in RU cells. Similar findings were also observed in MCF7 parental cells, which predominantly comprise RU cells (Additional file 1: Figure S2). As Sox2 did not induce an increase in the expression of Sox2 target genes in RU cells (Figure 3A), we hypothesized that the downregulation of Sox2 target genes in RU cells induced by Sox2 siRNA was mediated in a transcription-independent manner. If this is the case, the gene transcripts in RR cells are expected to be more sensitive to transcription inhibition than those in RU cells. In keeping with this concept, the addition of the transcription inhibitor, Triptolide, significantly decreased the transcript level of *PROM1* (CD133) in RR cells but paradoxically increased that in RU cells (Figure 3C).

### Mucin-15, a novel Sox2 target, contributes to mammosphere formation

**Figure 4:**
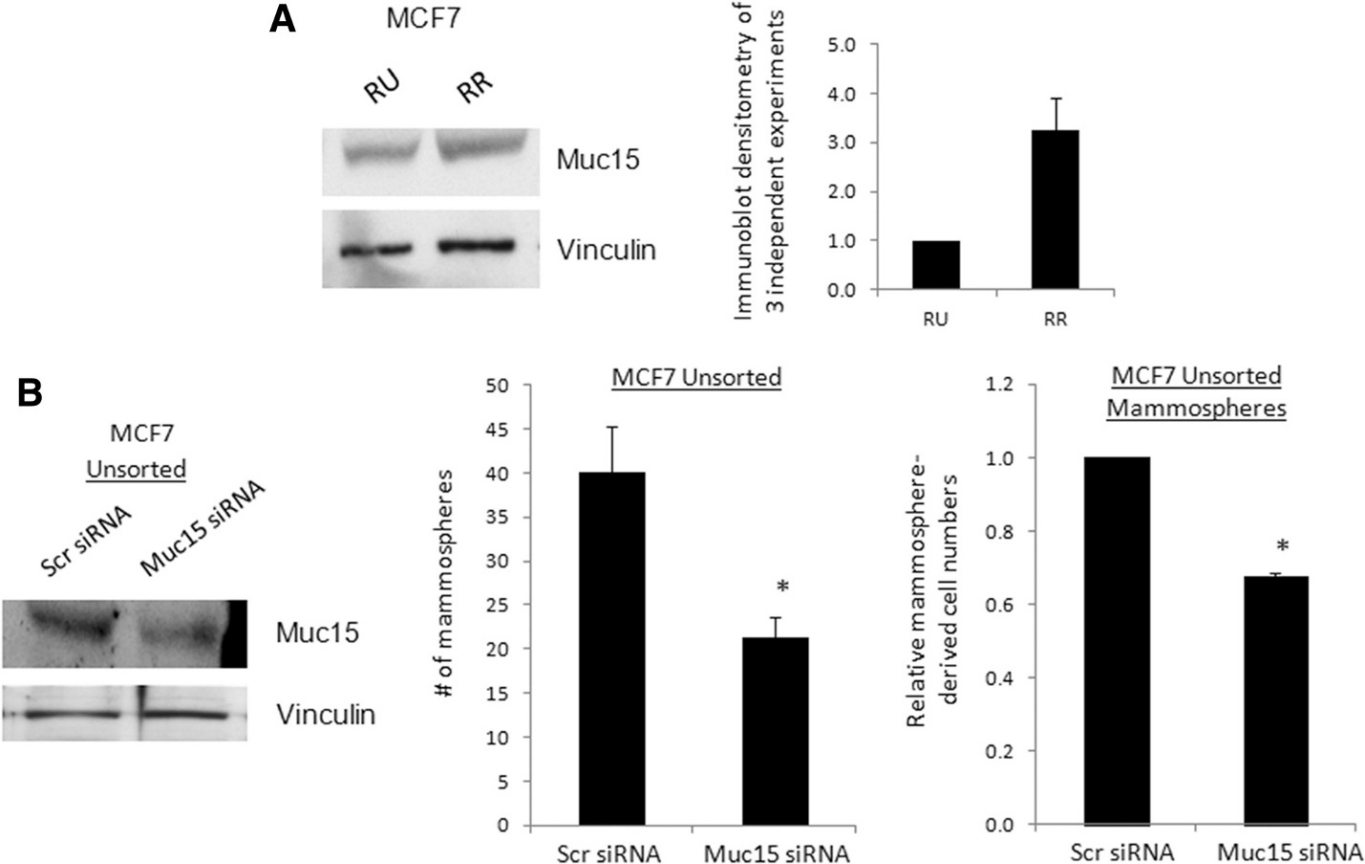
**Mammosphere formation is dependent on novel sex-determining region Y (SRY)-box binding protein-2 (Sox2) target Muc15. (A)** Western blot analysis of Muc15 in MCF7 reporter unrepsonisve (RU) and reporter responsive (RR) cells. **(B)** MCF7 unsorted cells were treated with 40 nM of Muc15 siRNA for 72 hours before seeding into mammosphere culture. Mammospheres were counted on day 7, and subsequently trypsinized and counted after trypan blue incubation. Accompanying western blot shows Muc15 knockdown efficiency.

To further support the concept that Sox2 contributes to tumorigenesis and stemness in BC by upregulating these stem cell- or cancer cell-associated genes, we examined the oncogenic effects of Mucin-15 (Muc15), which has not been previously shown to be a Sox2 downstream target. While Muc15 has been shown to play a key role in increasing invasiveness and tumorigenic capacity in colon cancer [47], it has not been linked to BC. As shown in Figures 2 and 4A, Muc15 was more highly expressed at the mRNA and protein levels in RR cells, as compared to RU cells. Furthermore, as shown above, overexpression or knockdown of Sox2 significantly modulated the expression of Muc15. As shown in Figure 4B, knockdown of Muc15 using siRNA significantly decreased the number of mammospheres formed from MCF7 unsorted cells, which comprise natural proportions of RU and RR subsets. Furthermore, using trypan blue exclusion assay, we found that siRNA knockdown of Muc15 significantly reduced the number of viable cells derived from the mammospheres (Figure 4B). The same experiment was repeated using four pooled unique siRNA sequences and we observed the same results, with Muc15 knockdown verified (Additional file 1: Figure S3).

### RR cells derived from primary patient breast tumors exhibit elevated tumorigenic properties and expression of target genes

**Figure 5:**
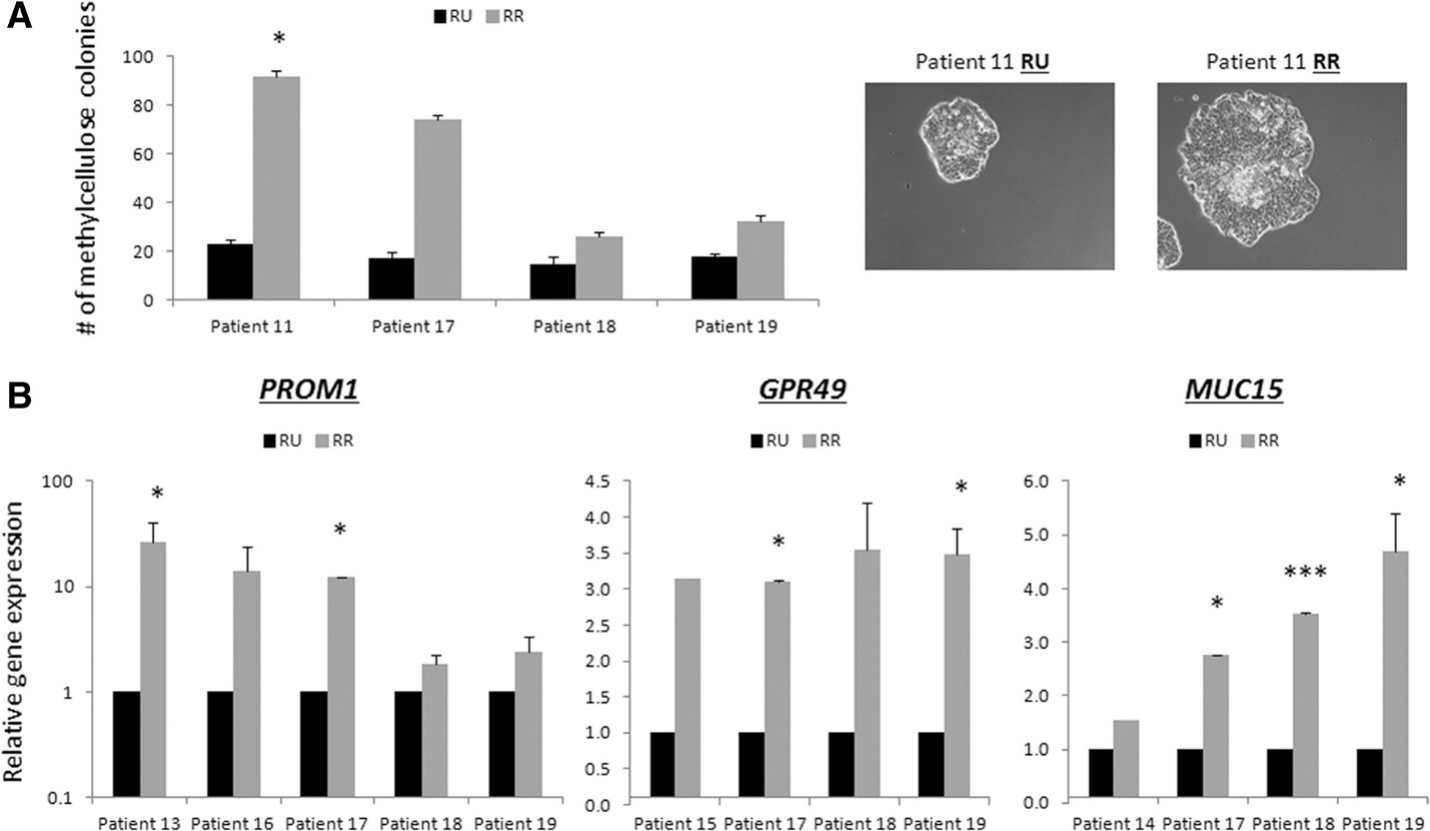
**Reporter responsive (RR) cells derived from primary patient breast tumors exhibit enhanced tumorigenic properties and elevated expression of target genes. (A)** Representative anchorage-independent methylcellulose colony formation assay numerical and pictorial results from patients 11, 17, 18, and 19 reporter unresponsive (RU) and reporter responsive (RR) cell populations. **(B)**. Quantitative-PCR *PROM1*, *MUC15*, and *GPR49* mRNA transcript analysis of fluorescence-activated cell sorting-purified lentiviral Sox2 transcription activity reported-infected primary patient breast tumor RU and RR cells from patients 13 to 19.

Lastly, we examined if BC cells derived from patient samples displayed similar findings to the MCF7 cells. Due to the relatively small number of tumor cells available, and the relatively low proportions of RR cells, we modified our Sox2 reporter such that it carried two signals, with the expression of RFP indicating successful infection with the viral vector, and the GFP signal indicating Sox2 reporter activity (detailed in main Methods section). Only cells expressing RFP but not GFP were regarded as RU cells, whereas those lacking both RFP and GFP were excluded from the analysis. Results from 19 primary BC tumors are summarized in Table 2. All 19 samples contained a detectable subset of RR cells, and the size of this population ranged from 0.3% to 23.8%. Interestingly, estrogen receptor-negative tumors (n = 3) had a significantly lower proportion of RR cells (p = 0.001). Functional studies were performed in eight samples in total. As shown in Figure 5A, RR cells were more efficient in forming colonies on methylcellulose agar in four out of four patient cells sampled. Importantly, as we gated our cells using RFP and GFP expression, we demonstrate that the RFP+ GFP- cells were healthy in culture (Additional file 1: Figure S4A-B). Under a fluorescence microscope, the cells were confirmed to be RFP+ (data not shown). As shown in Additional file 1: Figure S4B-C, RU and RR cells derived from patient samples had a similar Sox2 protein expression level in the nuclei, suggesting that the differences observed are not simply due to a lack of Sox2 protein or Sox2 nuclear localization in RU cells. Using fresh primary patient samples, we went on to test if RU and RR cells also differ in the expression of Sox2 downstream targets. Due to the relatively small number of primary samples available for testing, we chose three genes, including *PROM1* (CD133), *GPR49* (LGR5), and *MUC15*, based on the fact that the expression of these genes were amongst the most responsive to modulation of Sox2 (Figure 3A and B). As shown in Figure 5B, in a total of seven fresh primary patient samples, we detected higher expression of these three genes in patient RR cells as compared to their RU counterparts, although statistical analysis was not possible for all due to limitations in patient materials. Further, some patient samples did not contain enough RNA for analysis for all genes. Nevertheless, the overall findings from patient samples appear to mirror those in MCF7 cells.

**Table 2:** **RU and RR cell populations are detectable in primary patient breast tumors**

Patient number	Infection efficiency (RFP+)	RR cells (GFP+/RFP+), %	Nuclear Sox2 (IHC)	Estrogen receptor status
1	64.2%	13.9%	2+	+
2	48.7%	11.0%	2+	+
3	63.1%	16.1%	1+	+
4	57.8%	0.6%	N/A	-
5	43.0%	10.8%	N/A	+
6	49.7%	0.3%	3+	-
7	81.3%	12.5%	N/A	+
8	77.3%	21.4%	1+	+
9	36.3%	0.4%	N/A	-
10	61.0%	11.5%	N/A	+
11	- *	5.7%	0	+
12	- *	5.8%	3+	+
13	- *	17.0%	N/A	+
14	- *	21.6%	N/A	+
15	- *	23.8%	3+	+
16	- *	19.6%	2+	+
17	-*	5.4%	N/A	N/A
18	-*	10.5%	N/A	N/A
19	-*	5.8%	N/A	N/A

Flow cytometry analyses of red fluorescent protein (RFP) and green fluorescent protein (GFP) in mCMV-GFP-EF1-Puro infected primary breast tumor cells to set the gate thresholds and the SRR2-mCMV-GFP-EF1-RFP infected primary breast tumor cells showing % RFP+ cells (lentivirus infection efficiency) and % RFP+ GFP+ cells (% of patient reporter responsive (RR) cells). Table summarizes data from 19 primary breast tumor samples. The asterisks denote samples where the primary breast tumor cells % RFP+ could not be accurately assessed due to technical issues but is estimated to be approximately 60%. IHC denotes immunohistochemistry; N/A, not analyzed.

## Discussion

We recently identified two Sox2-expressing, phenotypically distinct cell subsets in BC cells, separated based on their differential response to a Sox2 transcription activity reporter, with RR cells showing higher tumorigenicity and more stem-like features relative to RU cells [18]. In the same study, we also found that these phenotypic differences are dependent on Sox2, as siRNA knockdown of Sox2 abrogates many of these phenotypic differences [18]. Because Sox2 is a transcription factor, we hypothesized that Sox2 contributes to the phenotypic differences between RU and RR cells by mediating differential gene expression. To test this hypothesis, we compared the Sox2 gene occupancy in RU cells with that of RR cells. Importantly, we found a largely mutually exclusive Sox2 promoter occupancy between these two cell subsets. Furthermore, there were a number of cancer- or stem-cell associated genes that are only found in the RR gene lists. Experiments using enforced expression or siRNA knockdown of Sox2 support the direct role of Sox2 in regulating these genes. The biological significance of our findings is supported by our results generated from the use of patient samples. Taken together, we believe that the overall findings lend support to our hypothesis.

Although aberrant Sox2 expression is well-documented in cancer, its mechanism of action in the regulation of downstream targets is incompletely understood. Currently, with the exception of *CCND1* (encoding Cyclin D1) [8], no other gene has been identified as a direct downstream target of Sox2 in BC. Nevertheless, a few Sox2 downstream gene targets have been reported in other cancer types, including *PROM1* (encoding CD133) in human lung cancer cells [48] and *ITGA6* (encoding CD49f) in human mesenchymal stem cells [49]. Regarding the functional importance of Sox2 in cancer, an exciting finding from our ChIP-chip study is that Sox2 was bound to the promoters of many cancer- and stem cell-associated genes in RR cells. This finding correlates well with the prevailing concept that the expression of embryonic stem cell markers in cancer cells results in stem-like features, which are often associated with an aggressive clinical course and treatment resistance [36],[50]. We believe that our finding of Sox2 regulating an array of cancer- and stem cell-associated genes provides a mechanistic explanation as to how Sox2 enhances stemness and tumorigenesis in cancer cell subsets. The importance of stem cell markers in identifying cancer stem cells, including Frizzled-4, Lgr5, and CD133 have previously been demonstrated [39],[51],[52], and here our data suggest that their expressions may be dependent on common precursor protein Sox2. Furthermore, we have demonstrated that CD133 and Lgr5 mRNA transcripts were also upregulated in primary tumor-derived RR cells from patients.

Importantly, it should be noted that the identification of our list of 15 novel Sox2 targets were hand-picked by us using a manual search approach. As we were most interested in better understanding how Sox2 contributes to BC and/or BC stem cell biology, we chose genes with published roles in that context. As a result, we have discovered that Sox2 does regulate an intriguing list of genes in the RR cells, but this does not exclude the possibility that other important cancer and/or stem cell genes exist in our ChIP-chip lists. Additionally, as the ChIP-chip assay is limited by the detection of hybridization of our Sox2-bound DNA samples to the microarray, our list certainly does not exhaust all the possible promoter interactions of Sox2 in BC cells. Importantly, we have done motif analyses on the ChIP-chip data, and have confirmed that Sox2 motifs previously published by others are enriched in our Sox2 ChIP DNA from both subsets [30].

We hypothesize that Sox2 in RU and RR cells are biochemically distinct, allowing for differential transcription activation ability at unique promoter regions. The RU cells exhibit no transcription activity as reported by our Sox2 reporter, and here we have shown that Sox2 overexpression did not transactivate the RR ChIP-chip promoters. Moreover, we have shown by ChIP-chip and conventional ChIP-PCR that Sox2 does not occupy the same promoters in RU and RR cells. These results suggest that Sox2 does not interact with these promoters in RU cells the same way as in RR cells. Conversely, Sox2 in RU cells binds to its own large cohort of gene promoters. This suggests multiple possibilities for the role of Sox2 at the RU gene promoters: 1) Sox2 could be suppressing gene expression of these genes as we have recently reported [53]; 2) Sox2 is transcriptionally active in RU cell gene promoters but did not transactivate luciferase or GFP expression from the reporter due to discrepancies between the reporter and gene promoters in Sox2 consensus binding sequences and/or adjacent sequences that can recruit other co-factors; 3) Sox2 occupancy at these promoters serves as a positive or negative facilitator to other transcriptional co-factor binding and/or activation; and 4) Sox2 is non-functional at these promoter regions due to an absent co-factor or post-translational modification that is present in RR cells.

While we found that Sox2 is directly involved in regulating the expression of its target genes in RR cells, the finding that siRNA knockdown of Sox2 decreased gene transcript expression in RU cells is a rather unexpected finding. From our previous studies, we found that Sox2 exists in the cytoplasm [18], and it can potentially carry out functions related to post-transcriptional modifications and/or translational modulations. One possible explanation is that Sox2 regulates the expression of these genes by non-transcriptional mechanisms. It is possible that Sox2 can prolong the integrity and half-life of specific gene transcripts, or it functions as a translation factor. To examine the contributions of transcriptional and non-transcriptional mechanisms in RU and RR cells, we used transcription inhibitor Triptolide. In RR cells, we found that Sox2 target *PROM1* transcripts were sensitive to the treatment and the mRNA levels decreased with increasing concentrations, supporting the hypothesis that Sox2 is transcriptionally activating *PROM1*. In RU cells, we did not observe decreased *PROM1* transcript levels with transcription inhibitor treatment, suggesting that Sox2 in RU cells may have a distinct regulatory mechanism for Sox2 target *PROM1*.

We have focused on Muc15 in our studies as it is a new putative onco-protein, consistently highly expressed in RR cells, and responsive to Sox2 regulation. In particular, Muc15 is of interest to us as it is relatively unknown in the cancer biology of any tissue. Muc15 is a highly glycosylated extracellular mucin protein previously reported to be expressed in normal epithelial cells, including the breast, but elevated in tumor cell populations [44],[47],[54]-[56]. In this report, we are the first to identify very high Muc15 expression in BC cells. Importantly, we detected increased Muc15 mRNA transcript and protein levels in RR cells compared to RU cells in cell lines and primary patient samples. Muc15 was previously demonstrated to promote oncogenesis in colon cancer cells *in vitro* and *in vivo* [47]. Thus, our ChIP-chip study is a good resource for novel putative therapeutic BC targets.

We showed that the patient RU and RR cells have distinct phenotypes as demonstrated in an anchorage-independent methylcellulose colony formation assay and underlying biology as determined by qPCR. Importantly, we have confirmed that RU cells from patients, although reporter unresponsive, do express nuclear Sox2 as detected by immunohistochemistry techniques. We have also uncovered potential mechanisms underlying the more tumorigenic RR cells as the patient RR cells exhibited higher expression levels of Sox2 target genes, *PROM1*, *GPR49*, and *MUC15* transcripts. Thus, the response of BC cells to the Sox2 transcription activity reporter has distinguished primary patient and cultured cell lines cancer-cell subpopulations with distinct phenotypic and molecular features.

## Conclusions

**Figure 6:**
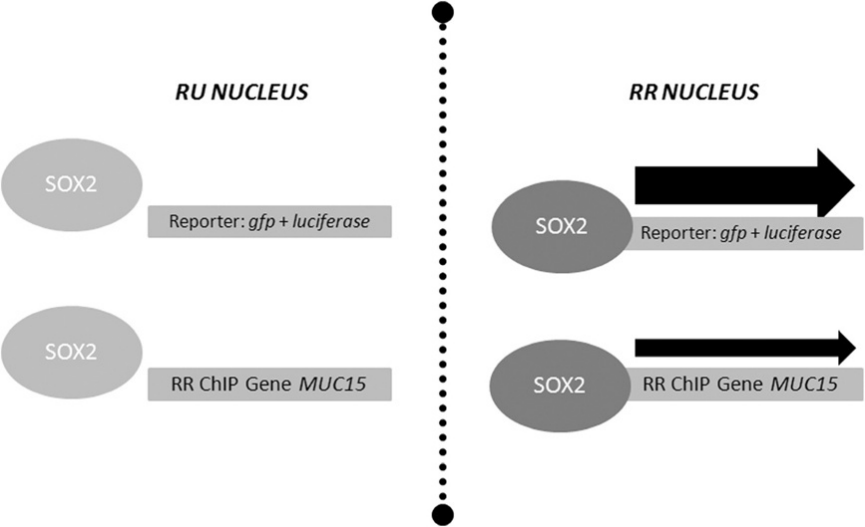
**Schematic diagram of sex-determining region Y (SRY)-box binding protein-2 (Sox2) transcription activity heterogeneity model in breast cancer cells.** Our working model depicts that the Sox2 in reporter responsive (RR) cells is distinct from that of reporter unresponsive (RU) cells and confers high transcription activity in that subset perhaps partially through differential interactions with promoter DNA in the nucleus. ChIP, chromatin immunoprecipitation.

Taken together, we have shown that Sox2 behaves heterogeneously in breast tumor cell populations. Sox2 is strongly bound to a subset of cancer and stem cell gene promoters and can upregulate the corresponding gene transcripts in RR cells but not in RU cells. Importantly, we have identified a novel Sox2 target Muc15 that is important for mammosphere formation, and is also upregulated in the tumorigenic RR cells derived from primary patient breast tissue samples. In summary, we depict in a schematic diagram where Sox2 in RR cells interacts with DNA, and/or transcriptionally activates promoters differently compared to Sox2 in RU cells (Figure 6).

## Authors’ contributions

KJ conceived and designed the research plan, optimized and performed the chromatin immunoprecipitation, analyzed the microarray results, performed all the cell line work and experiments, assisted with some of the patient sample work and experiments, analyzed data, and wrote the manuscript. PW, NG, KG, FW, and XY performed the majority of the patient sample work and experiments. AA provided assistance in performing and analyzing the qPCR experiments. GB and TPM collected the patient tumours, provided clinical data, and gave intellectual input. BSA provided intellectual input and critical reading of the manuscript. RL conceived and designed the research plan and wrote the manuscript. All authors read and approved the final manuscript.

## Additional files

## Electronic supplementary material

Additional file 1: Supplementary Materials and Methods, and Figures S1, S2, S3, and S4. (DOCX 1 MB)

Additional file 2: Table S1.: Primer sequences used for chromatin immunoprecipitation (ChIP)-PCR and quantitative (q)-PCR. All ChIP-PCR primers were designed using Primer 3. All q-PCR primers were designed using PrimerBank, Massachusetts General Hospital, Harvard Medical School (http://pga.mgh.harvard.edu/primerbank/). (XLSX 11 KB)

Additional file 3: Table S2.: MCF7 reporter unresponsive (RU) and reporter responsive (RR) gene lists with chromatin immunoprecipitation (ChIP)-chip promoter microarray peak score >2.0-fold compared to input DNA (1830 + 456 genes). (XLSX 141 KB)

Below are the links to the authors’ original submitted files for images.

Authors’ original file for figure 1

Authors’ original file for figure 2

Authors’ original file for figure 3

Authors’ original file for figure 4

Authors’ original file for figure 5

Authors’ original file for figure 6

## Abbreviations

BC: breast cancer
bp: base pairs
BSA: bovine serum albumin
ChIP: chromatin immunoprecipitation
ChIP-chip: chromatin immunoprecipitation promoter microarray chip analysis
ER: estrogen receptor
FACS: fluorescence-activated cell sorting
FBS: fetal bovine serum
GFP: green fluorescent protein
Muc15: mucin-15
PANTHER: Protein Analysis THrough Evolutionary Relationships
qPCR: quantitative reverse transcription polymerase chain reaction
RFP: red fluorescent protein
RR: reporter responsive
RU: reporter unresponsive
Sox2: sex-determining region Y (SRY)-box binding protein-2
SRR2: Sox2 regulatory region 2

## References

[1] Takahashi K, Tanabe K, Ohnuki M, Narita M, Ichisaka T, Tomoda K, Yamanaka S (2007). Induction of pluripotent stem cells from adult human fibroblasts by defined factors. Cell.

[2] Boyer LA, Lee TI, Cole MF, Johnstone SE, Levine SS, Zucker JP, Guenther MG, Kumar RM, Murray HL, Jenner RG, Gifford DK, Melton DA, Jaenisch R, Young RA (2005). Core transcriptional regulatory circuitry in human embryonic stem cells. Cell.

[3] Sarkar A, Hochedlinger K (2013). The sox family of transcription factors: versatile regulators of stem and progenitor cell fate. Cell Stem Cell.

[4] Arnold K, Sarkar A, Yram MA, Polo JM, Bronson R, Sengupta S, Seandel M, Geijsen N, Hochedlinger K (2011). Sox2(+) adult stem and progenitor cells are important for tissue regeneration and survival of mice. Cell Stem Cell.

[5] Roy S, Gascard P, Dumont N, Zhao J, Pan D, Petrie S, Margeta M, Tlsty TD (2013). Rare somatic cells from human breast tissue exhibit extensive lineage plasticity. Proc Natl Acad Sci U S A.

[6] Wang Y, Dong J, Li D, Lai L, Siwko S, Li Y, Liu M (2013). Lgr4 regulates mammary gland development and stem cell activity through the pluripotency transcription factor Sox2. Stem Cells.

[7] Leis O, Eguiara A, Lopez-Arribillaga E, Alberdi MJ, Hernandez-Garcia S, Elorriaga K, Pandiella A, Rezola R, Martin AG (2012). Sox2 expression in breast tumors and activation in breast cancer stem cells. Oncogene.

[8] Chen Y, Shi L, Zhang L, Li R, Liang J, Yu W, Sun L, Yang X, Wang Y, Zhang Y, Shang Y (2008). The molecular mechanism governing the oncogenic potential of SOX2 in breast cancer. J Biol Chem.

[9] Rudin CM, Durinck S, Stawiski EW, Poirier JT, Modrusan Z, Shames DS, Bergbower EA, Guan Y, Shin J, Guillory J, Rivers CS, Foo CK, Bhatt D, Stinson J, Gnad F, Haverty PM, Gentleman R, Chaudhuri S, Janakiraman V, Jaiswal BS, Parikh C, Yuan W, Zhang Z, Koeppen H, Wu TD, Stern HM, Yauch RL, Huffman KE, Paskulin DD, Illei PB (2012). Comprehensive genomic analysis identifies SOX2 as a frequently amplified gene in small-cell lung cancer. Nat Genet.

[10] Annovazzi L, Mellai M, Caldera V, Valente G, Schiffer D (2011). SOX2 expression and amplification in gliomas and glioma cell lines. Cancer Genomics Proteomics.

[11] Zhang J, Chang DY, Mercado-Uribe I, Liu J (2012). Sex-determining region Y-box 2 expression predicts poor prognosis in human ovarian carcinoma. Hum Pathol.

[12] Basu-Roy U, Seo E, Ramanathapuram L, Rapp TB, Perry JA, Orkin SH, Mansukhani A, Basilico C (2012). Sox2 maintains self renewal of tumor-initiating cells in osteosarcomas. Oncogene.

[13] Neumann J, Bahr F, Horst D, Kriegl L, Engel J, Luque RM, Gerhard M, Kirchner T, Jung A (2011). SOX2 expression correlates with lymph-node metastases and distant spread in right-sided colon cancer. BMC Cancer.

[14] Laga AC, Zhan Q, Weishaupt C, Ma J, Frank MH, Murphy GF (2011). SOX2 and nestin expression in human melanoma: an immunohistochemical and experimental study. Exp Dermatol.

[15] Lengerke C, Fehm T, Kurth R, Neubauer H, Scheble V, Muller F, Schneider F, Petersen K, Wallwiener D, Kanz L, Fend F, Perner S, Bareiss PM, Staebler A (2011). Expression of the embryonic stem cell marker SOX2 in early-stage breast carcinoma. BMC Cancer.

[16] Hagerstrand D, He X, Bradic Lindh M, Hoefs S, Hesselager G, Ostman A, Nister M (2011). Identification of a SOX2-dependent subset of tumor- and sphere-forming glioblastoma cells with a distinct tyrosine kinase inhibitor sensitivity profile. Neuro Oncol.

[17] Nakatsugawa M, Takahashi A, Hirohashi Y, Torigoe T, Inoda S, Murase M, Asanuma H, Tamura Y, Morita R, Michifuri Y, Kondo T, Hasegawa T, Takahashi H, Sato N (2011). SOX2 is overexpressed in stem-like cells of human lung adenocarcinoma and augments the tumorigenicity. Lab Invest.

[18] Wu F, Zhang J, Wang P, Ye X, Jung K, Bone KM, Pearson JD, Ingham RJ, McMullen TP, Ma Y, Lai R (2012). Identification of two novel phenotypically distinct breast cancer cell subsets based on Sox2 transcription activity. Cell Signal.

[19] Zhang Y, Eades G, Yao Y, Li Q, Zhou Q (2012). Estrogen receptor alpha signaling regulates breast tumor-initiating cells by down-regulating miR-140 which targets the transcription factor SOX2. J Biol Chem.

[20] Singh S, Trevino J, Bora-Singhal N, Coppola D, Haura E, Altiok S, Chellappan SP (2012). EGFR/Src/Akt signaling modulates Sox2 expression and self-renewal of stem-like side-population cells in non-small cell lung cancer. Mol Cancer.

[21] Lin CW, Liao MY, Lin WW, Wang YP, Lu TY, Wu HC (2012). Epithelial cell adhesion molecule regulates tumor initiation and tumorigenesis via activating reprogramming factors and epithelial-mesenchymal transition gene expression in colon cancer. J Biol Chem.

[22] Chen YL, Wang SY, Liu RS, Wang HE, Chen JC, Chiou SH, Chang CA, Lin LT, Tan DT, Lee YJ (2012). Remnant living cells that escape cell loss in late-stage tumors exhibit cancer stem cell-like characteristics. Cell Death Dis.

[23] Han X, Fang X, Lou X, Hua D, Ding W, Foltz G, Hood L, Yuan Y, Lin B (2012). Silencing SOX2 induced mesenchymal-epithelial transition and its expression predicts liver and lymph node metastasis of CRC patients. PLoS One.

[24] Lu X, Deng Q, Li H, Suo Z (2011). Altered characteristics of cancer stem/initiating cells in a breast cancer cell line treated with persistent 5-FU chemotherapy. Exp Ther Med.

[25] Wang X, Liang Y, Chen Q, Xu HM, Ge N, Luo RZ, Shao JY, He Z, Zeng YX, Kang T, Yun JP, Xie F (2012). Prognostic significance of SOX2 expression in nasopharyngeal carcinoma. Cancer Invest.

[26] Ben-Porath I, Thomson MW, Carey VJ, Ge R, Bell GW, Regev A, Weinberg RA (2008). An embryonic stem cell-like gene expression signature in poorly differentiated aggressive human tumors. Nat Genet.

[27] Gomez-Mateo Mdel C, Piqueras M, Pahlman S, Noguera R, Navarro S (2011). Prognostic value of SOX2 expression in neuroblastoma. Genes Chromosomes Cancer.

[28] Sholl LM, Barletta JA, Yeap BY, Chirieac LR, Hornick JL (2010). Sox2 protein expression is an independent poor prognostic indicator in stage I lung adenocarcinoma. Am J Surg Pathol.

[29] Simoes BM, Piva M, Iriondo O, Comaills V, Lopez-Ruiz JA, Zabalza I, Mieza JA, Acinas O, Vivanco MD (2011). Effects of estrogen on the proportion of stem cells in the breast. Breast Cancer Res Treat.

[30] Tomioka M, Nishimoto M, Miyagi S, Katayanagi T, Fukui N, Niwa H, Muramatsu M, Okuda A (2002). Identification of Sox-2 regulatory region which is under the control of Oct-3/4-Sox-2 complex. Nucleic Acids Res.

[31] O'Geen H, Nicolet CM, Blahnik K, Green R, Farnham PJ (2006). Comparison of sample preparation methods for ChIP-chip assays. Biotechniques.

[32] Spandidos A, Wang X, Wang H, Seed B (2010). PrimerBank: a resource of human and mouse PCR primer pairs for gene expression detection and quantification. Nucleic Acids Res.

[33] Wang X, Spandidos A, Wang H, Seed B (2012). PrimerBank: a PCR primer database for quantitative gene expression analysis, 2012 update. Nucleic Acids Res.

[34] Zhang J, Wang P, Wu F, Li M, Sharon D, Ingham RJ, Hitt M, McMullen TP, Lai R (2012). Aberrant expression of the transcriptional factor Twist1 promotes invasiveness in ALK-positive anaplastic large cell lymphoma. Cell Signal.

[35] Mi H, Muruganujan A, Thomas PD (2013). PANTHER in 2013: modeling the evolution of gene function, and other gene attributes, in the context of phylogenetic trees. Nucleic Acids Res.

[36] Medema JP (2013). Cancer stem cells: the challenges ahead. Nat Cell Biol.

[37] Alison MR, Islam S, Wright NA (2010). Stem cells in cancer: instigators and propagators?. J Cell Sci.

[38] Gupta S, Iljin K, Sara H, Mpindi JP, Mirtti T, Vainio P, Rantala J, Alanen K, Nees M, Kallioniemi O (2010). FZD4 as a mediator of ERG oncogene-induced WNT signaling and epithelial-to-mesenchymal transition in human prostate cancer cells. Cancer Res.

[39] Jin X, Jeon HY, Joo KM, Kim JK, Jin J, Kim SH, Kang BG, Beck S, Lee SJ, Kim JK, Park AK, Park WY, Choi YJ, Nam DH, Kim H (2011). Frizzled 4 regulates stemness and invasiveness of migrating glioma cells established by serial intracranial transplantation. Cancer Res.

[40] Nielsen TO, Andrews HN, Cheang M, Kucab JE, Hsu FD, Ragaz J, Gilks CB, Makretsov N, Bajdik CD, Brookes C, Neckers LM, Evdokimova V, Huntsman DG, Dunn SE (2004). Expression of the insulin-like growth factor I receptor and urokinase plasminogen activator in breast cancer is associated with poor survival: potential for intervention with 17-allylamino geldanamycin. Cancer Res.

[41] Oakes SR, Naylor MJ, Asselin-Labat ML, Blazek KD, Gardiner-Garden M, Hilton HN, Kazlauskas M, Pritchard MA, Chodosh LA, Pfeffer PL, Lindeman GJ, Visvader JE, Ormandy CJ (2008). The Ets transcription factor Elf5 specifies mammary alveolar cell fate. Genes Dev.

[42] Chen D, Bhat-Nakshatri P, Goswami C, Badve S, Nakshatri H (2013). ANTXR1, a stem cell-enriched functional biomarker, connects collagen signaling to cancer stem-like cells and metastasis in breast cancer. Cancer Res.

[43] Chaudhary A, Hilton MB, Seaman S, Haines DC, Stevenson S, Lemotte PK, Tschantz WR, Zhang XM, Saha S, Fleming T, St Croix B (2012). TEM8/ANTXR1 blockade inhibits pathological angiogenesis and potentiates tumoricidal responses against multiple cancer types. Cancer Cell.

[44] Pallesen LT, Berglund L, Rasmussen LK, Petersen TE, Rasmussen JT (2002). Isolation and characterization of MUC15, a novel cell membrane-associated mucin. Eur J Biochem.

[45] Oskarsson T, Acharyya S, Zhang XH, Vanharanta S, Tavazoie SF, Morris PG, Downey RJ, Manova-Todorova K, Brogi E, Massague J (2011). Breast cancer cells produce tenascin C as a metastatic niche component to colonize the lungs. Nat Med.

[46] Medeiros RB, Papenfuss KJ, Hoium B, Coley K, Jadrich J, Goh SK, Elayaperumal A, Herrera JE, Resnik E, Ni HT (2009). Novel sequential ChIP and simplified basic ChIP protocols for promoter co-occupancy and target gene identification in human embryonic stem cells. BMC Biotechnol.

[47] Huang J, Che MI, Huang YT, Shyu MK, Huang YM, Wu YM, Lin WC, Huang PH, Liang JT, Lee PH, Huang MC (2009). Overexpression of MUC15 activates extracellular signal-regulated kinase 1/2 and promotes the oncogenic potential of human colon cancer cells. Carcinogenesis.

[48] Iida H, Suzuki M, Goitsuka R, Ueno H (2012). Hypoxia induces CD133 expression in human lung cancer cells by up-regulation of OCT3/4 and SOX2. Int J Oncol.

[49] Yu KR, Yang SR, Jung JW, Kim H, Ko K, Han DW, Park SB, Choi SW, Kang SK, Scholer H, Kang KS (2012). CD49f enhances multipotency and maintains stemness through the direct regulation of OCT4 and SOX2. Stem Cells.

[50] Apostolou P, Toloudi M, Chatziioannou M, Ioannou E, Papasotiriou I (2012). Cancer stem cells stemness transcription factors expression correlates with breast cancer disease stage. Curr Stem Cell Res Ther.

[51] Sato T, Clevers H (2013). Growing self-organizing mini-guts from a single intestinal stem cell: mechanism and applications. Science.

[52] Singh SK, Hawkins C, Clarke ID, Squire JA, Bayani J, Hide T, Henkelman RM, Cusimano MD, Dirks PB (2004). Identification of human brain tumor initiating cells. Nature.

[53] Wu F, Ye X, Wang P, Jung K, Wu C, Douglas D, Kneteman N, Bigras G, Ma Y, Lai R (2013). Sox2 suppresses the invasiveness of breast cancer cells via a mechanism that is dependent on Twist1 and the status of Sox2 transcription activity. BMC Cancer.

[54] Wang RY, Chen L, Chen HY, Hu L, Li L, Sun HY, Jiang F, Zhao J, Liu GM, Tang J, Chen CY, Yang YC, Chang YX, Liu H, Zhang J, Yang Y, Huang G, Shen F, Wu MC, Zhou WP, Wang HY (2013). MUC15 inhibits dimerization of EGFR and PI3K-AKT signaling and is associated with aggressive hepatocellular carcinomas in patients. Gastroenterology.

[55] Nam KH, Noh TW, Chung SH, Lee SH, Lee MK, Hong SW, Chung WY, Lee EJ, Park CS (2011). Expression of the membrane mucins MUC4 and MUC15, potential markers of malignancy and prognosis, in papillary thyroid carcinoma. Thyroid.

[56] Riker AI, Enkemann SA, Fodstad O, Liu S, Ren S, Morris C, Xi Y, Howell P, Metge B, Samant RS, Shevde LA, Li W, Eschrich S, Daud A, Ju J, Matta J (2008). The gene expression profiles of primary and metastatic melanoma yields a transition point of tumor progression and metastasis. BMC Med Genomics.

