# Supplementary material for: Profiling gene promoter occupancy of Sox2 in two phenotypically distinct breast cancer cell subsets using chromatin immunoprecipitation and genome-wide promoter microarrays
Source: Breast Cancer Res. 2014 Nov 8;16:470. doi: 10.1186/s13058-014-0470-2 (PMC4303205; doi:10.1186/s13058-014-0470-2)
Supplement: Supplementary file 1 — Additional file 1: Supplementary Materials and Methods, and Figures S1, S2, S3, and S4. (DOCX 1 MB) [file 13058_2014_470_MOESM1_ESM.docx]

**ADDITIONAL FILE 1 - Jung et al. Profiling gene promoter occupancy of Sox2 in two phenotypically distinct breast cancer cell subsets using chromatin immunoprecipitation and genome-wide promoter microarrays.**

**Supplementary Materials and Methods**

***ChIP-chip microarray and analyses***

We performed chromatin immunoprecipitation (ChIP) on purified MCF7 RU and RR cells, isolated and amplified the DNA, and submitted the duplicated DNA samples, MCF7 RU IgG and Sox2 ChIP, MCF7 RR IgG and Sox2 ChIP, and MCF7 RU and RR input DNA, to Roche Nimblegen ChIP-chip array services for quality assessment and experimental analysis. The DNA samples were hybridized to the Roche Nimblegen Human ChIP-chip 3x720K RefSeq Promoter array, with probe length of 50-75 bp, median probe spacing of 100 bp, and promoter tiling ranging from -3200 to +800 relative to the transcription start site (TSS). Using a manufacturer specified stringent threshold of peak scores >2.0 (compared to input DNA signal) and false discovery rate (FDR) of <0.05, we discovered Sox2 was bound to the promoter regions of 1830 unique genes in RU cells and 456 unique genes in RR cells with an overlap of 62 genes between the two subsets (3.5% and 15.7% respectively). These gene targets were considered the "highest-confidence protein binding sites", as defined by the microarray manufacturer.

We shortlisted the ChIP-chip analyses provided by Roche Nimblegen services using the following criteria. First, only feature peak scores (microarray signal strength) >2.0 (compared to input DNA signal) and feature peak FDR (false discovery rate) <0.05 were included. Second, we shortlisted the microarray features to only those found in both replicates (approximately 80% replicate overlaps for RU and RR subsets), and we averaged their feature peak scores. Third, if the same feature was found in the IgG pulldown (<1%), the IgG feature peak score was subtracted from the averaged Sox2 peak score. We ranked the remaining features by their new adjusted peak scores and listed the unique genes.

We functionally annotated the protein classes of the gene lists using the online software, www.pantherdb.org.

**Supplementary Figures**

**
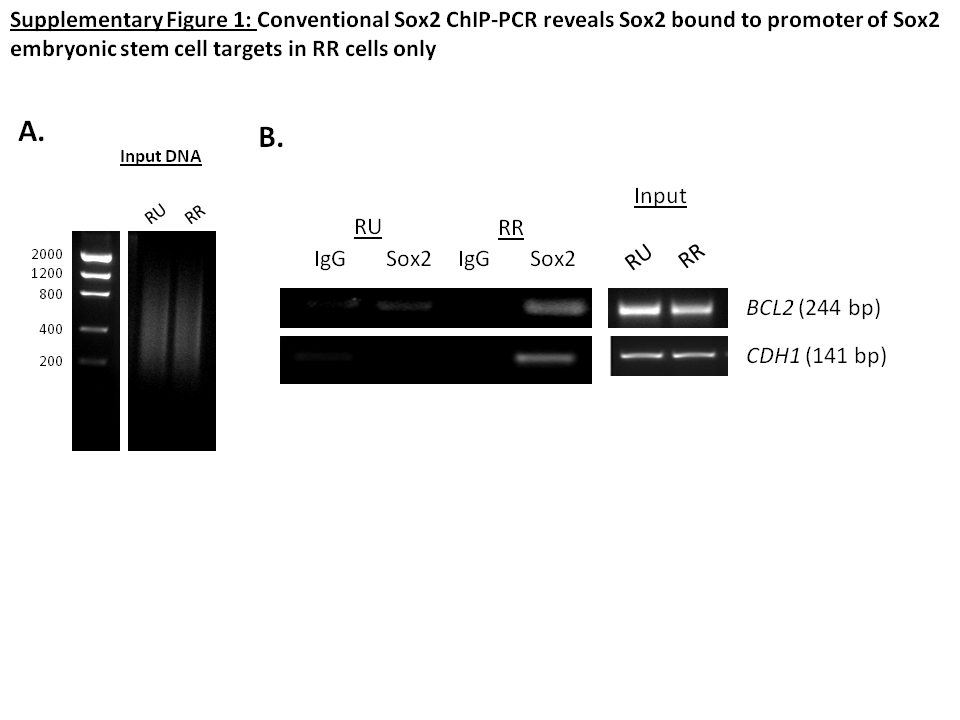

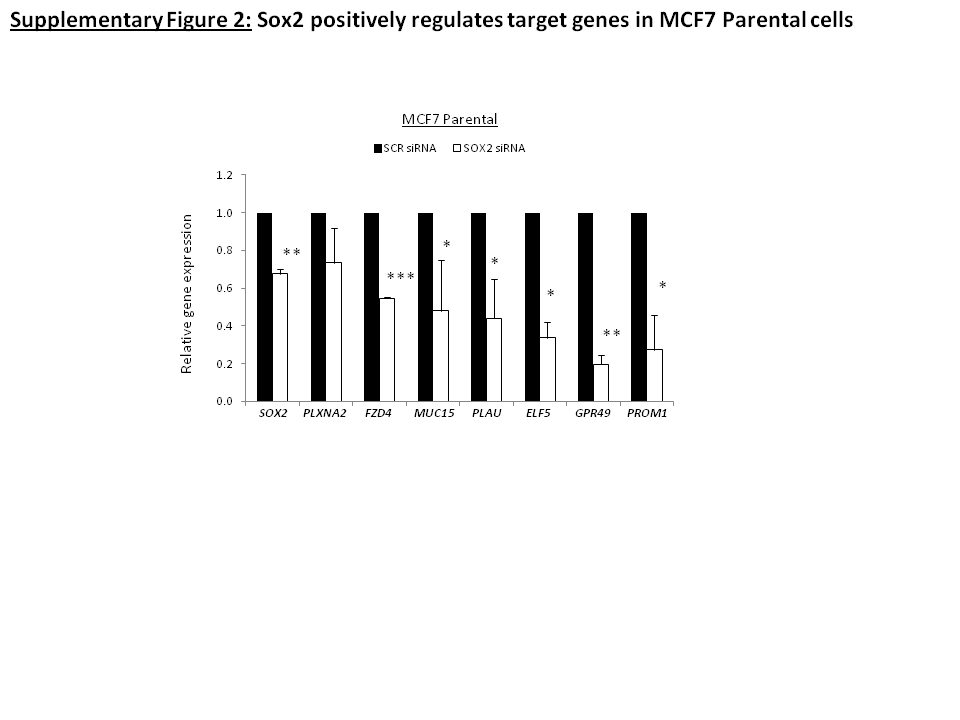

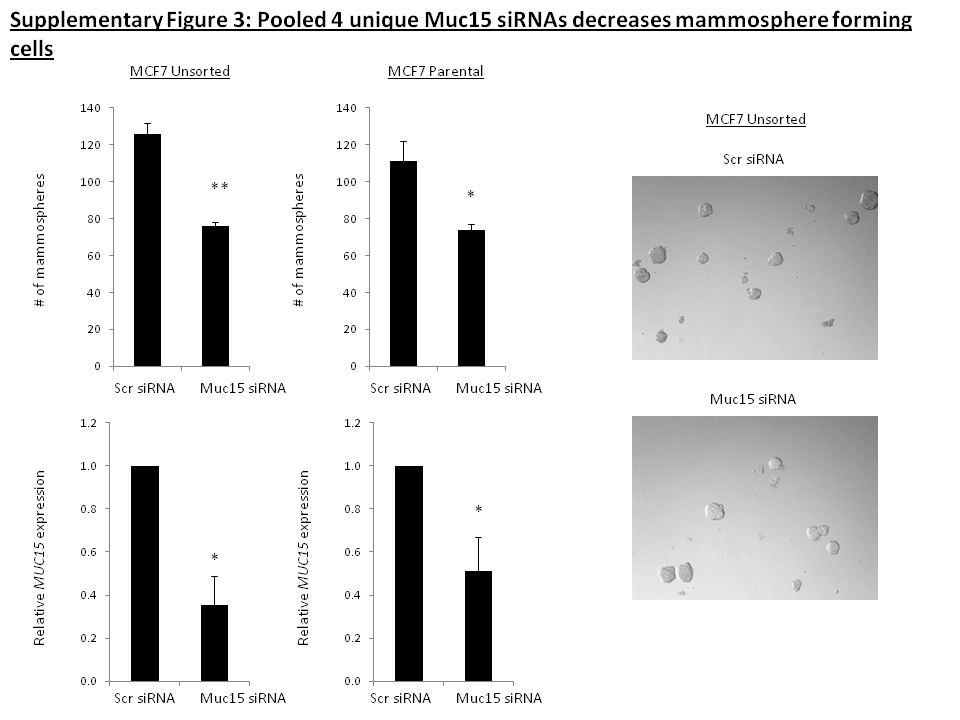

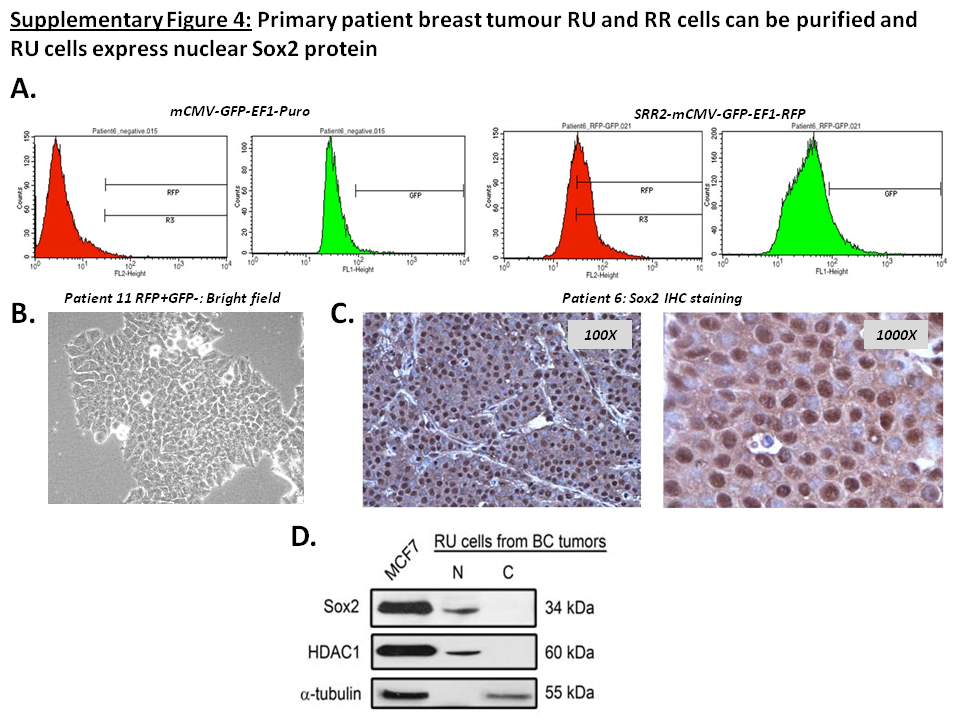
**
