# Supplementary figures and images for: Profiling gene promoter occupancy of Sox2 in two phenotypically distinct breast cancer cell subsets using chromatin immunoprecipitation and genome-wide promoter microarrays
Source: Breast Cancer Res. 2014 Nov 8;16:470. doi: 10.1186/s13058-014-0470-2 (PMC4303205; doi:10.1186/s13058-014-0470-2)

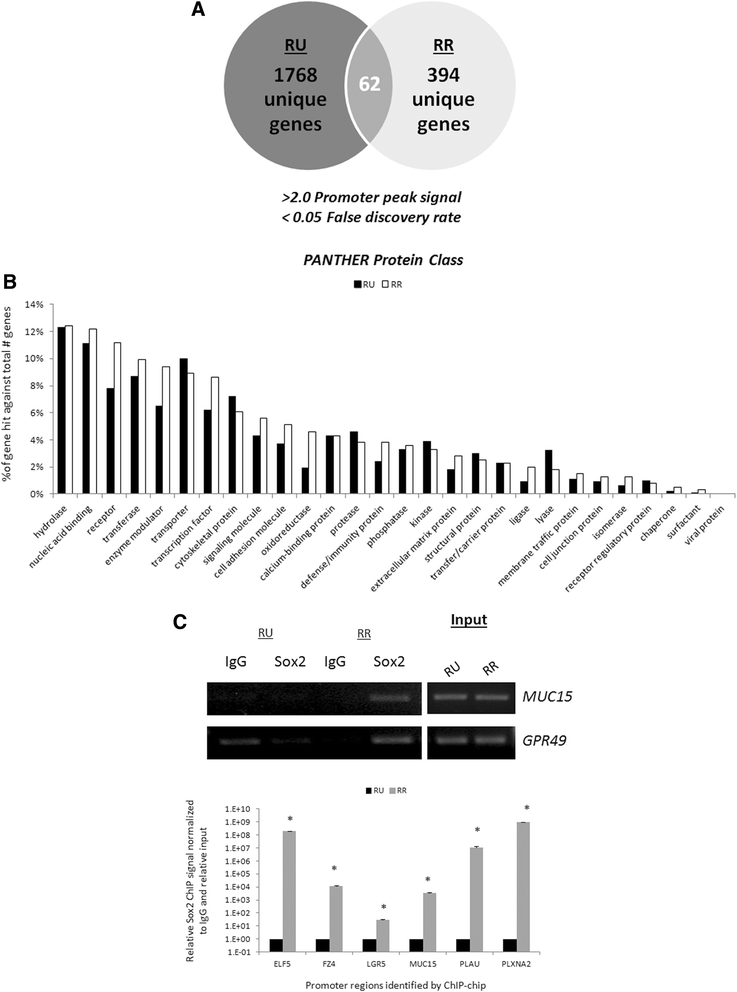

Supplement: Supplementary file 4 — Authors’ original file for figure 1 [file 13058_2014_470_MOESM4_ESM.gif]

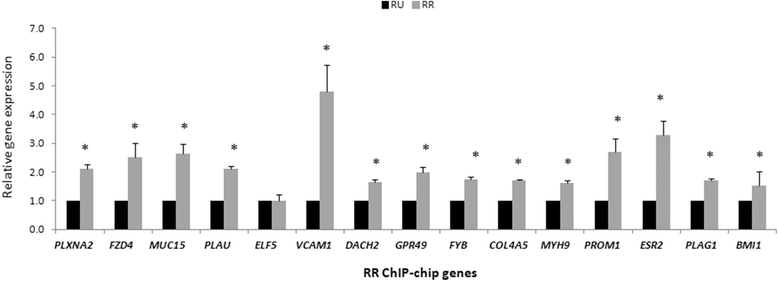

Supplement: Supplementary file 5 — Authors’ original file for figure 2 [file 13058_2014_470_MOESM5_ESM.gif]

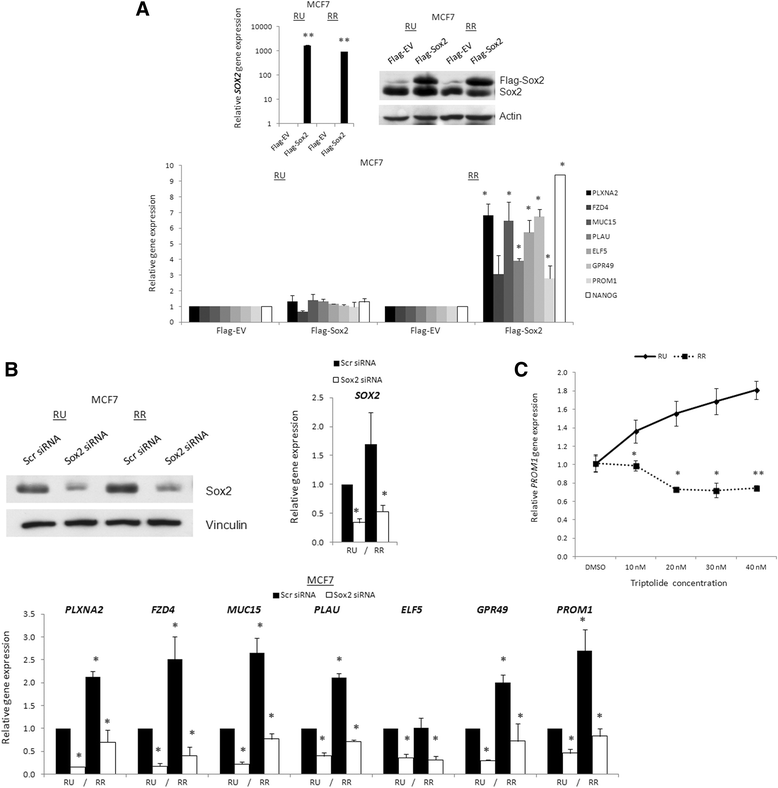

Supplement: Supplementary file 6 — Authors’ original file for figure 3 [file 13058_2014_470_MOESM6_ESM.gif]

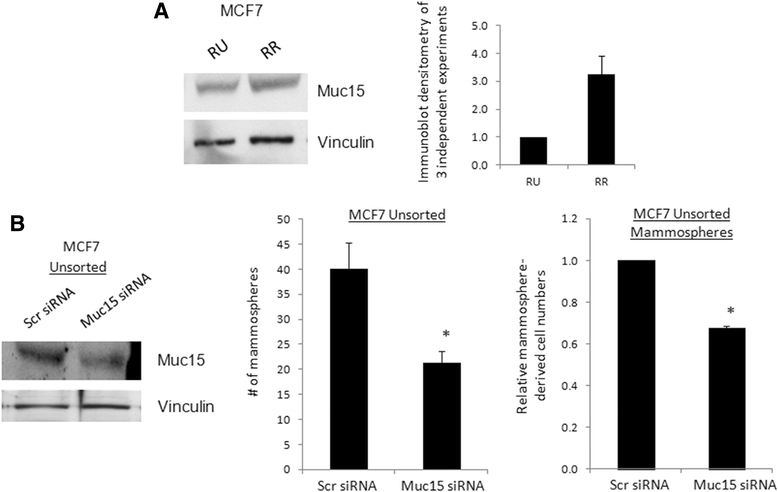

Supplement: Supplementary file 7 — Authors’ original file for figure 4 [file 13058_2014_470_MOESM7_ESM.gif]

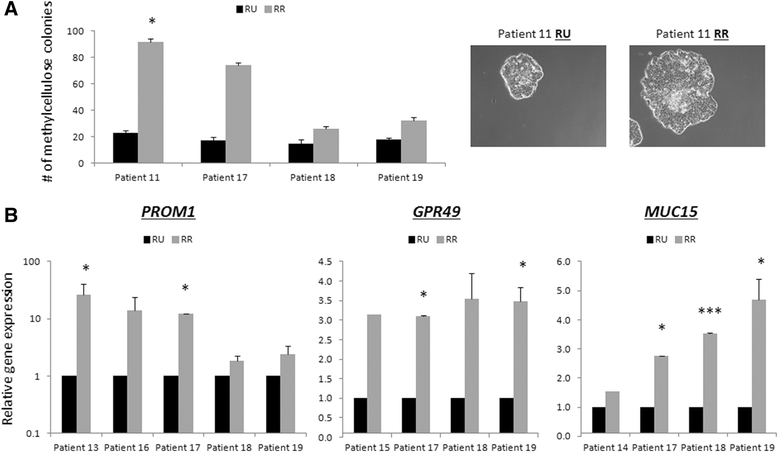

Supplement: Supplementary file 8 — Authors’ original file for figure 5 [file 13058_2014_470_MOESM8_ESM.gif]

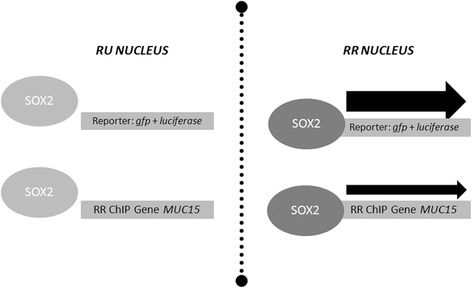

Supplement: Supplementary file 9 — Authors’ original file for figure 6 [file 13058_2014_470_MOESM9_ESM.gif]
